# PyPLIF HIPPOS-Assisted Prediction of Molecular Determinants of Ligand Binding to Receptors

**DOI:** 10.3390/molecules26092452

**Published:** 2021-04-22

**Authors:** Enade P. Istyastono, Nunung Yuniarti, Vivitri D. Prasasty, Sudi Mungkasi

**Affiliations:** 1 Faculty of Pharmacy, Sanata Dharma University, Yogyakarta 55282, Indonesia; 2 Department of Pharmacology and Clinical Pharmacy, Faculty of Pharmacy, Universitas Gadjah Mada, Yogyakarta 55281, Indonesia; nunung@mail.ugm.ac.id; 3 Faculty of Biotechnology, Atma Jaya Catholic University of Indonesia, Jakarta 12930, Indonesia; vivitri.dewi@atmajaya.ac.id; 4 Department of Mathematics, Faculty of Science and Technology, Sanata Dharma University, Yogyakarta 55282, Indonesia; sudi@usd.ac.id

**Keywords:** PyPLIF HIPPOS, AutoDock Vina, drug discovery, fragment-based, molecular determinant, G protein-coupled receptor

## Abstract

Identification of molecular determinants of receptor-ligand binding could significantly increase the quality of structure-based virtual screening protocols. In turn, drug design process, especially the fragment-based approaches, could benefit from the knowledge. Retrospective virtual screening campaigns by employing AutoDock Vina followed by protein-ligand interaction fingerprinting (PLIF) identification by using recently published PyPLIF HIPPOS were the main techniques used here. The ligands and decoys datasets from the enhanced version of the database of useful decoys (DUDE) targeting human G protein-coupled receptors (GPCRs) were employed in this research since the mutation data are available and could be used to retrospectively verify the prediction. The results show that the method presented in this article could pinpoint some retrospectively verified molecular determinants. The method is therefore suggested to be employed as a routine in drug design and discovery.

## 1. Introduction

Information on the important amino acid residues that bind to ligand could significantly increase the quality of structure-based drug design and discovery, especially in computer-aided fragment-based approaches [1]. The application of the knowledge in structure-based virtual screening (SBVS) campaigns has led to successful discoveries of novel fragments targeting histamine H_1_ [2], H_3_ [3], and H_4_ [4] receptors. The studies employed the previously identified Asp107 [5,6,7,8,9], Asp114 [5,7,8,9], and Asp94 [5,7,8,9,10] as the molecular determinants of the ligand binding to the histamine H_1_, H_3_, and H_4_ receptors, respectively. The SBVS campaigns have benefited from more than 20 years of mutagenesis studies on G protein-coupled receptors (GPCRs) [5,8,9,11]. However, not all drug targets have the privileges that the GPCRs have.

Development of computational methods to accurately identify molecular determinants of the receptor-ligand binding is of considerable interest. Istyastono et al. [12] combined three-dimension (3D) QSAR analysis and molecular docking simulations to pinpoint the molecular determinants in histamine H_4_ receptor-ligand binding. The results were confirmed by site-directed mutagenesis (SDM) studies and identified Asn147, Glu182, Thr323, and Gln347 as the molecular determinants [12]. On the other hand, Istyastono et al. [13] employed a combination of molecular docking simulations using PLANTS [14], protein-ligand interaction fingerprinting (PLIF) using PyPLIF [15,16], and supervised machine learning using Recursive Partition and Regression Trees (RPART) [17] in a retrospective SBVS campaign targeting estrogen receptor alpha (ERα) to optimize the prediction quality. Besides being able to optimize the prediction quality of the SBVS protocol, the combined methods provided information on some probable molecular determinants in the receptor-ligand binding [13]. Unfortunately, unlike for GPCRs, there are no such comprehensive mutation data for ERα. The mutation data collected in GPCRdb [8,9] offer possibilities to retrospectively verify the identified molecular determinants.

The upgraded version of PyPLIF called PyPLIF HIPPOS was recently made publicly available [18]. The software offers some new features to perform a similar technique introduced by Istyastono et al. [13] in more efficient ways [18]. The research presented in this manuscript aimed to introduce and retrospectively verify the computational techniques to identify the molecular determinants of the ligand binding to the receptors by employing a combination of molecular docking simulations using AutoDock Vina [19], PLIF using PyPLIF HIPPOS [18], and supervised machine learning using RPART [13,17] in retrospective SBVS campaigns targeting Adenosine A_2a_ receptor (AA2AR), β_2_ adrenergic receptor (ADRB2), C-X-C chemokine receptor type 4 (CXCR4), and Dopamine D3 receptor (DRD3). These receptors were selected because they are members of GPCRs, their ligands and decoys datasets are available in the enhanced version of the database of useful decoys (DUDE) [20], and the crystal structures of the human receptors are available.

## 2. Results

**Figure 1:**
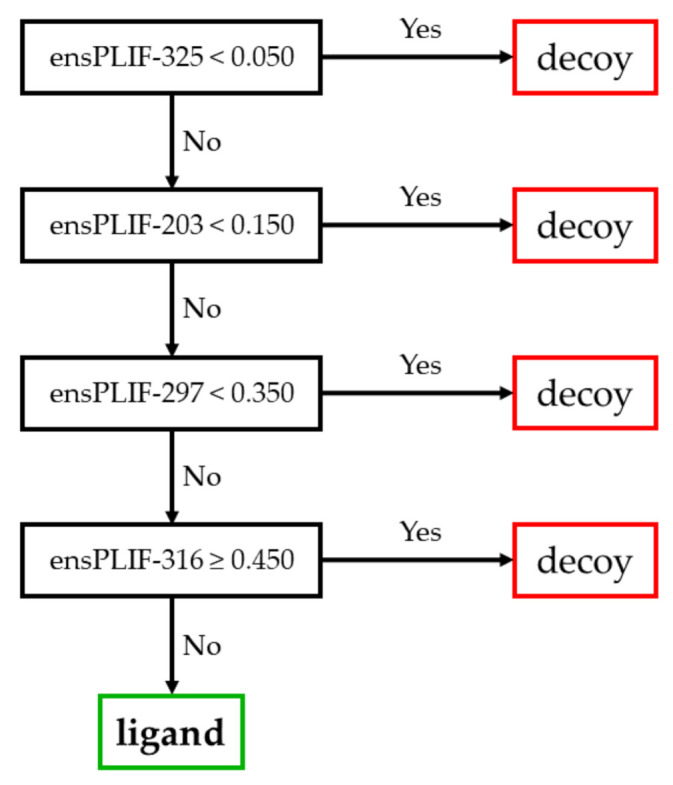
The best decision tree to identify ligand for A2AR.

**Figure 2:**
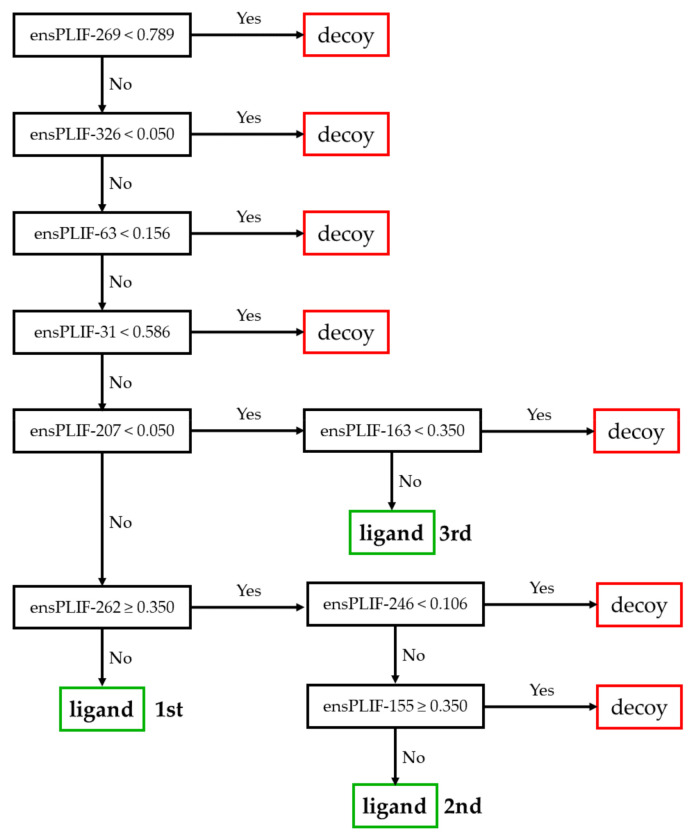
The best decision tree to identify ligand for ADRB2.

**Figure 3:**
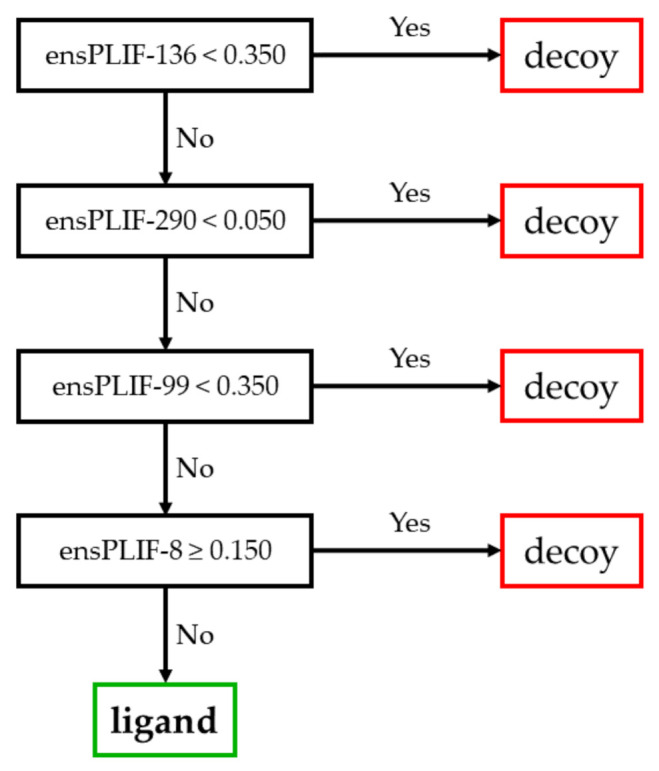
The best decision tree to identify ligand for CXCR4.

**Figure 4:**
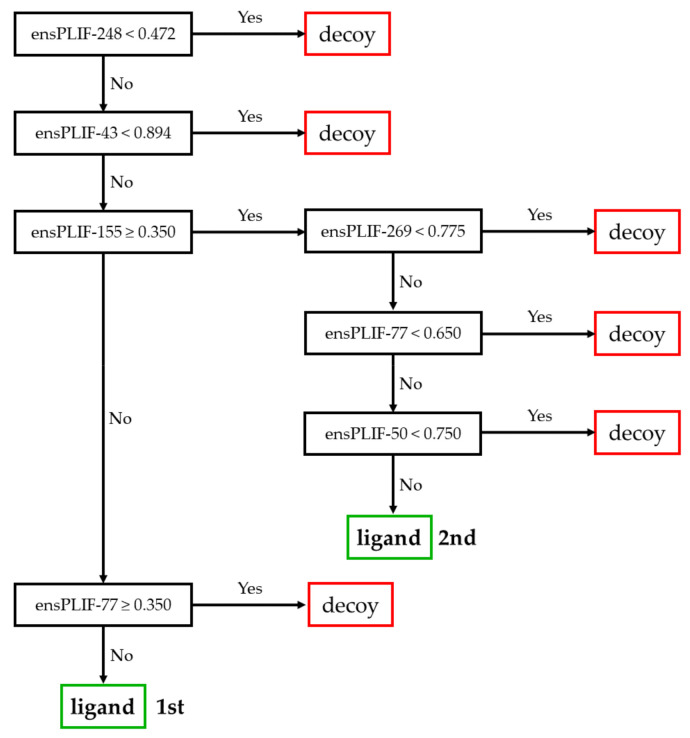
The best decision tree to identify ligand for DRD3.

The proposed method identified 23 probable molecular determinants of the ligand binding to the studied receptors (Table 1). Thirteen out of these 23 molecular determinants were verified by examining the mutation data in GPCRdb [5,11]. The molecular determinants were four, nine, four, and six amino acid residues identified as the important residues of the ligand binding to A2AR, ADRB2, CXCR4, and DRD3, respectively. Those residues were related to five out of seven protein-ligand interaction types identified using PyPLIF HIPPOS [18]. The highest frequency of the essential interaction was aromatic edge-to-face interaction (nine occurrences), followed by hydrophobic interaction (eight occurrences), ionic interaction with the residues as the anion (three occurrences), aromatic face-to-face interaction (two occurrences), and hydrogen-bond (h-bond) with the residues as the donor (two occurrences). There was no h-bond with the residue as the acceptor, nor the ionic interaction with the residue as the cation identified as the important interaction in this study. The residues presented in Table 1 were extracted from the best decision trees resulted from retrospective SBVS campaigns targeting the studied receptors. The best decision trees to retrospectively identify ligand or decoy for AA2AR, ADRB2, CXCR4, and DRD3 are presented in Figure 1, Figure 2, Figure 3, and Figure 4, respectively. The decision trees were resulted from RPART analysis using ensemble PLIF (ensPLIF) as the descriptor (vide infra; Materials and Methods).

### 2.1. The Best Decision Tree Related to AA2AR 

In Figure 1, there is one branch leading to ligand identification. There are four ensPLIF descriptors that play an important role, i.e., ensPLIF-203, -297, -316, and -325. In AA2AR, these ensPLIF descriptors related to the ionic interaction with the residue Glu203 as the anion, the aromatic edge-to-face interaction to Trp246, the hydrophobic interaction to Leu249, and the aromatic edge-to-face interaction to His250, respectively (Table 1). The decision tree indicates that the hydrophobic interaction to Leu249 is unfavorable.

### 2.2. The Best Decision Tree Related to ADRB2 

In Figure 2, there are three branches leading to ligand identification. There are nine ensPLIF descriptors that play an important role, i.e., ensPLIF-31, -63, -155, -163, -207, -246, -262, -269, and -326. In ADRB2, these ensPLIF descriptors related to the aromatic edge-to-face interaction to Trp109, the ionic interaction with Asp113 as the anion, the hydrophobic interaction to Asp192, the aromatic face-to-face interaction to Phe193, the h-bond with Ser204 as the donor, the hydrophobic interaction to Trp286, the aromatic edge-to-face interaction to Phe289, the aromatic edge-to-face interaction to Phe290, and the h-bond with Asn312 as the donor, respectively (Table 1). The decision tree indicates that the aromatic edge-to-face interaction to Phe289 and the hydrophobic interaction to Asp192 are unfavorable interactions.

### 2.3. The Best Decision Tree Related to CXCR4 

Similar to Figure 1, there is one branch leading to ligand identification in Figure 3. There are four ensPLIF descriptors that play an important role, i.e., ensPLIF-8, -99, -136, and -290. In CXCR4, these ensPLIF descriptors related to the hydrophobic interaction to Glu32, the hydrophobic interaction to Asp97, the aromatic edge-to-face interaction to Trp102, and the aromatic edge-to-face interaction to Tyr255, respectively (Table 1). The decision tree indicates that the hydrophobic interaction to Glu32 is unfavorable.

### 2.4. The Best Decision Tree Related to DRD3 

Two branches are leading to ligand identification in Figure 4. There are six ensPLIF descriptors that play an important role, i.e., ensPLIF-43, -50, -77, -155, -248, and -269. In DRD3, these ensPLIF descriptors related to the hydrophobic interaction to Phe106, the hydrophobic interaction to Val107, the ionic interaction with Asp 110 as the anion, the hydrophobic interaction to Ile183, the aromatic edge-to-face interaction to Phe346, and the aromatic edge-to-face interaction to His349, respectively (Table 1). The decision tree indicates that the ionic interaction with Asp 110 as the anion and the hydrophobic interaction to Ile183 could serve as unfavorable interactions.

## 3. Discussion

The proposed computational methods presented in this article (vide infra; Materials and Methods) predicted in total 23 molecular determinants of the ligand binding to some GPCRs, i.e., AA2AR, ADRB2, CXCR4, and DRD3. Thirteen out of these 23 molecular determinants (circa 56.52%) were retrospectively verified by observing the mutant data compiled in GPCRdb (https://gpcrdb.org/) (accessed on 20 March 2021) [8,9]. There are still 10 predicted molecular determinants that expectantly could be verified in the near future. Notably, the EF values as the prediction quality indicators of the SBVS protocols outperformed the original SBVS protocols published in [20], which is also an indication that the use of AutoDock Vina was reliable in the SBVS campaigns.

The computational techniques employing the combination of retrospective SBVS campaign and RPART analysis were originally used to increase the prediction quality of the developed SBVS to identify ligand for ERα [22]. Subsequently, the descriptor ensPLIF was introduced to cover all relevant docking poses produced during the SBVS campaign in order to mimic the lock-and-key and the induced-fit theories [23] in the RPART analysis [13,24]. The protocols were then employed to prospectively screen and design novel ligands for ERα [13,25,26] and inhibitors for acetylcholine esterase (AChE) [27,28,29]. Interestingly, besides increasing the prediction quality of the SBVS protocols to identify ligand for ERα [13] and inhibitor for acetylcholine esterase (AChE) [24], the combined methods could also predict the important amino acid residues that play an essential role in the ligand binding to the proteins [13,24]. However, there was no database to retrospectively verify the prediction. One should perform SDM studies to have the verification of the predicted molecular determinants of the ligand binding.

The most recent GPCRdb updates have just been published [8], which also cover recent SDM studies on GPCRs [9]. On the other hand, PyPLIF [16] was also recently upgraded to PyPLIF HIPPOS [18], which was reported 10 times faster than its predecessor. PyPLIF HIPPOS provides a new feature to neglect the interactions to the backbone of the protein [18], which offers us to mimic SDM studies in silico. By employing the feature, the interactions to the backbones will no longer interfere with the RPART analysis, which in turn could avoid the emergence of strange interactions in the decision trees, e.g., h-bonds to Leu346, Ala350, and Gly420 in ERα [13]. Together with GPCRdb [8,9], PyPLIF HIPPOS [18] could be employed in the combination of retrospective SBVS campaign and RPART analysis similar to those performed in [13] to identify and retrospectively verify the molecular determinants of the ligand binding to GPCRs in full in silico studies. At the beginning of the studies, we found that the published version of PyPLIF HIPPOS [18] could not recognize the disulfide bridge in the protein, which was subsequently fixed in the 0.1.2 version. Therefore, PyPLIF HIPPOS version 0.1.2 was then employed throughout these studies. The compounds to perform retrospective SBVS campaigns were obtained from commonly used benchmarking datasets provided by DUDE [20]. As described previously, only GPCRs with human crystal structures used in DUDE were used in this study, i.e., A2AR, ADRB2, CXCR4, and DRD3 [20]. Instead of PLANTS docking software [14,30] used by [13,24], the currently popular docking software AutoDock Vina [19] was used in this study since PyPLIF HIPPOS provides a new feature to identify PLIF resulted from AutoDock Vina [18]. Similar to [13,24], the machine learning method RPART analysis was used in this study. The machine learning RPART was chosen here to avoid the usage of black-box methods [31]. On the contrary, the RPART analysis could provide information to pinpoint the probable molecular determinants of the ligand binding to the relevant receptors (Table 1). Notably, overfitting, cross-correlation, and chance correlation were not observed in all decision trees during the RPART analysis [13,32]. The ensPLIF values resulted from the retrospective SBVS campaigns are provided as Table S1 in the Supplementary Materials in case there will be further studies employing the data, e.g., optimizing the prediction quality of the SBVS protocols or employing other machine learning approaches for comparison.

The information of the molecular determinants could be used further in structure-based drug design and discovery, especially in fragment-based approaches to perform optimization rationally in order to fine-tune the affinity and the selectivity for a particular receptor target [33]. For example, the discovery of Gln347 of the histamine H_4_ receptor (HRH4) as the molecular determinant of the ligand binding could be used further to fine-tune the HRH4 affinity and the selectivity toward the histamine H_3_ receptor (HRH3) [12]. In the previous attempts targeting AChE, Phe331 was identified in silico as the molecular determinant of the ligand binding [24]. The information was used to design some chalcone derivatives and could discover in vitro potent chalcone derivatives as AChE inhibitors [24,27]. In our lab, the described method in this article is currently employed to design and discover novel ligands for the matrix metalloproteinase 9 (MMP9) [34,35] and dipeptidyl peptidase-4 (DPP4) [36,37].

### 3.1. The Identified Molecular Determinants of the Ligand Binding to AA2AR

All the identified molecular determinants in this receptor were verified in the GPCRdb (Table 1). Only one branch leading to ligand identification in the decision tree is presented in Figure 1, which indicates that the molecular determinants are ligand-independent. Interestingly, the decision tree indicates also that the hydrophobic interaction to Leu249 is an unfavorable interaction. This is difficult to verify since negative results are usually not being published. Fortunately, although the F-measure value (0.184) is slightly outperformed by the originally SBVS protocol (0.233) [20], the EF value (272.286) is significantly better compared to the original SBVS protocol (21.8) [20]. Moreover, the prediction quality of the SBVS could still be optimized by filtering poses based on the corresponding docking score [13,24].

### 3.2. The Identified Molecular Determinants of the Ligand Binding to ADRB2

Three out of nine identified molecular determinants in this receptor were verified in the GPCRdb (Table 1). There are three branches leading to ligand identification in the decision tree presented in Figure 2, which indicate that some of the molecular determinants are ligand-dependent. In this receptor, the aromatic edge-to-face interaction to Phe289 and the hydrophobic interaction to Asp192 are suggested as unfavorable interactions. The EF value of 465.151 and the F-measure value of 0.307 are significantly better compared to the original SBVS protocol (EF value = 3.9; F-measure value = 0.046) [20].

### 3.3. The Identified Molecular Determinants of the Ligand Binding to CXCR4

Three out of four identified molecular determinants in this receptor were verified in the GPCRdb (Table 1). Similar to AA2AR, there is only one branch leading to ligand identification in the decision tree presented in Figure 3, which indicates that the molecular determinants are ligand-independent. In this receptor, the hydrophobic interaction to Glu32 is an unfavorable interaction. The infinity EF value and the F-measure value of 0.307 are significantly better compared to the original SBVS protocol (EF value = 17.5; F-measure value = 0.280) [20].

### 3.4. The Identified Molecular Determinants of the Ligand Binding to DRD3

Three out of six identified molecular determinants in this receptor were verified in the GPCRdb (Table 1). There are two branches leading to ligand identification in the decision tree presented in Figure 4, which indicate that some of the molecular determinants are ligand-dependent. In this receptor, the ionic interaction with Asp 110 as the anion and the hydrophobic interaction to Ile183 could serve as unfavorable interactions. The EF value of 455.652 and the F-measure value of 0.169 outperform the original SBVS protocol (EF value = 4.4; F-measure value = 0.052) [20].

## 4. Materials and Methods

### 4.1. Materials

The computational simulations were performed in a 64-bit Linux (CentOS Linux release 7.4.1708) machine with Intel^®^ Xeon^®^ CPU E5-2620 v4 @ 2.10GHz as the processor and 64GB of random-access memory (RAM). There were in total 16 central processing units (CPUs) from 8 cores @ 2 threads. The following were the software used in the research presented in this article: AutoDock Vina version 1.1.2 [19]; PyPLIF HIPPOS [18] version 0.1.2 (https://github.com/radifar/PyPLIF-HIPPOS/releases/tag/0.1.2, accessed on 20 March 2021); PLANTS docking software 1.2 [14,30]; SPORES 1.3 [38]; Open Babel 2.4.1 [39]; ADFRsuite 1.0 [40]; and RPART package [17] in R statistical computing software version 3.6.0 [41]. Compound datasets for performing the retrospective SBVS campaigns were obtained from DUDE [20]. The crystal structures of the studied receptors were obtained from The Research Collaboratory for Structural Bioinformatics Protein Data Bank (RCSB PDB; https://www.rcsb.org/) (accessed on 20 March 2021) [42] with the PDB IDs of 3EML, 3NY8, 3ODU, and 3PBL for AA2AR, ADRB2, CXCR4, and DRD3, respectively. The crystal structures were the ones used by DUDE since the SBVS campaigns presented here were benchmarked to the ones presented in the publication [20]. The mutant data compiled in GPCRdb (https://gpcrdb.org/) (accessed on 20 March 2021) [8,9] were used for retrospective verification of the identified molecular determinants.

### 4.2. Methods

#### 4.2.1. Generic Procedure

The generic procedure consisted of 3 steps: (i) retrospective SBVS campaigns using AutoDock Vina; (ii) PLIF identification using PyPLIF HIPPOS followed by ensPLIF calculation; and (iii) RPART analysis using R.

**Figure 5:**
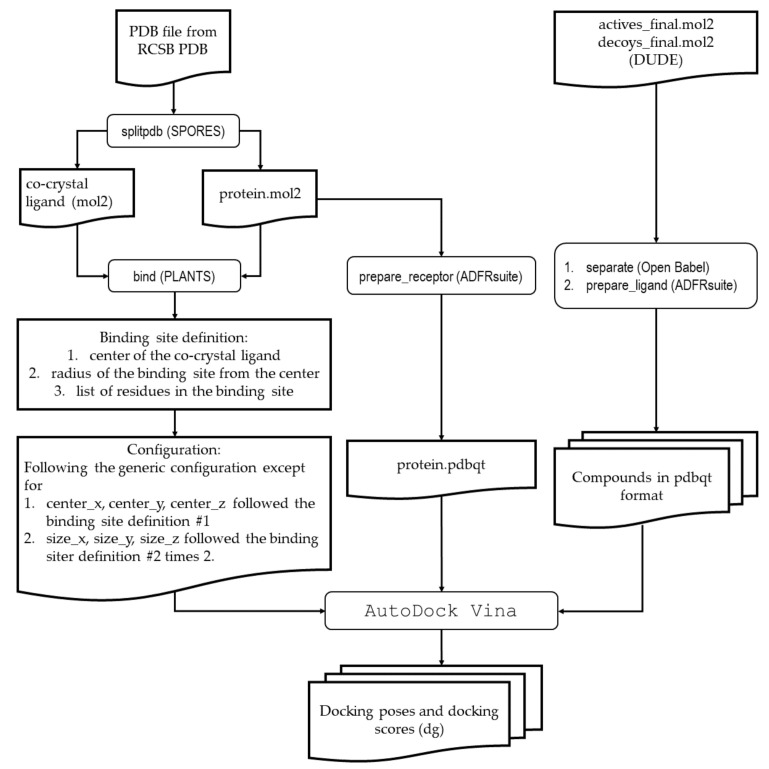
The procedure of the retrospective SBVS campaign.

The retrospective SBVS using molecular docking simulations started with the preparation of the compounds obtained as mol2 files from DUDE, followed by preparation of the virtual target obtained from RCSB PDB, and preparation of the configuration file to perform docking. Then, the docking simulations were performed for all prepared compounds. Module “separate” from Open Babel was used to split the obtained files from DUDE into a single file for a single compound. The mol2 files were then subjected to the module “prepare_ligand” from ADFRsuite to be converted into the AutoDock Vina readily format pdbqt. The module “splitpdb” from SPORES was used to split the protein part from others into the pdb files obtained from RCSB PDB. The “protein.mol2” resulted from the module “splitpdb” of SPORES was then subjected to the module “prepare_receptor” from ADFRsuite to be converted into the AutoDock Vina readily format pdbqt. The generic configuration for the docking simulations were set as follows: num_modes = 10; energy_range = 5; cpu = 8; log = out.log. The XYZ coordinate position and the size of the docking box were specific for each virtual target. The center of the co-crystal ligand was set as the XYZ coordinate position, and the distance of 5 Å from the surface of the co-crystal was used to calculate the docking box size. The module “bind” from PLANTS was used to obtain the values of the XYZ coordinate positions and the size of the docking boxes. Each prepared compound was then docked using AutoDock Vina. Figure 5 presents the procedure of the retrospective SBVS.

**Figure 6:**
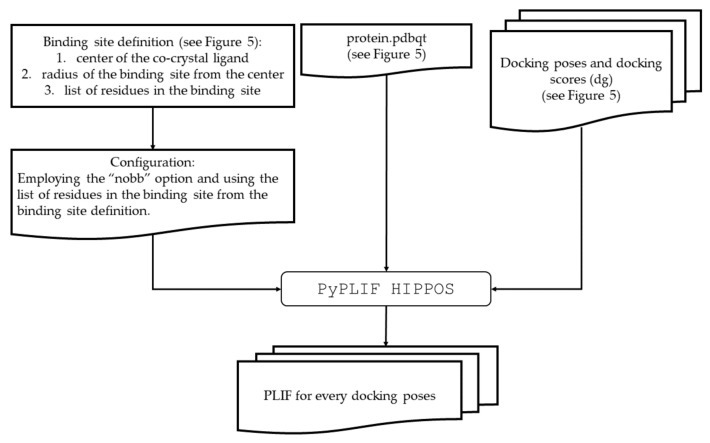
The procedure of the protein-ligand interaction fingerprinting (PLIF) identifications.

**Figure 7:**
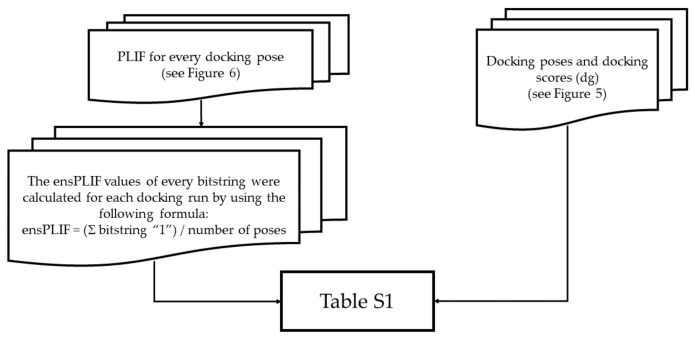
The calculation of ensPLIF [13] and the creation of Table S1.

The module “bind” from PLANTS also provided a list of residues in the docking box. The residues were used to create configuration files for PLIF identification using PyPLIF HIPPOS. By employing the configuration files, the PLIF identifications were performed for all docking poses resulted from the retrospective SBVS (Figure 6). The option “nobb” to neglect the interaction to the backbone atoms of the protein was used [18]. Subsequently, employing the similar procedure presented by [13], ensPLIF values were calculated (Figure 7). The results were then arranged in a table for each receptor to be easily analyzed using the RPART package in R (Table S1). The tables started with the first column named “y” encoding the observed data (“1” for active; “0” for decoy), followed by “name” for the name of the corresponding ligand, “dg” for the best affinity value resulted from the docking simulations for each compound, and then ensPLIF variables (“V1” for ensPLIF-1, “V2” for ensPLIF-2, until the whole ensPLIF values were covered for each receptor). The best decision trees resulted from the RPART analysis were then examined for possibilities of overfitting, the cross-correlation between identified ensPLIF variables, and chance-correlation [13,32].

#### 4.2.2. Identification of Molecular Determinant of Ligand Binding to AA2AR

The human AA2AR with the PDB ID of 3EML was downloaded from https://www.rcsb.org/structure/3eml (accessed on 20 March 2021) [42], while the corresponding actives_final.mol2 and decoys_final.mol2 were obtained from http://dude.docking.org/targets/aa2ar (accessed on 20 March 2021) [20]. Before performing the module “splitpdb” using SPORES, by employing the Unix grep command, only chain A was extracted from the 3eml.pdb for further analysis. The subsequent procedure followed the generic procedure (see Section 4.2.1).

#### 4.2.3. Identification of Molecular Determinant of Ligand Binding to ADRB2

The human ADRB2 with the PDB ID of 3NY8 was downloaded from https://www.rcsb.org/structure/3ny8 (accessed on 20 March 2021) [42], while the corresponding actives_final.mol2 and decoys_final.mol2 were obtained from http://dude.docking.org/targets/adrb2 (accessed on 20 March 2021) [20]. Similar to AA2AR (see Section 4.2.2), only chain A was employed in this study. The subsequent procedure followed the generic procedure (see Section 4.2.1).

#### 4.2.4. Identification of Molecular Determinant of Ligand Binding to CXCR4

The human CXCR4 with the PDB ID of 3ODU was downloaded from https://www.rcsb.org/structure/3odu (accessed on 20 March 2021) [42], while the corresponding actives_final.mol2 and decoys_final.mol2 were obtained from http://dude.docking.org/targets/cxcr4 (accessed on 20 March 2021) [20]. Unlike AA2AR and ADRB2, the whole crystal structure was employed in this study since the Unix grep command could not be used to extract chain A from this particular PDB format. The subsequent procedure followed the generic procedure (see Section 4.2.1).

#### 4.2.5. Identification of Molecular Determinant of Ligand Binding to DRD3

The human DRD3 with the PDB ID of 3PBL was downloaded from https://www.rcsb.org/structure/3pbl (accessed on 20 March 2021) [42], while the corresponding actives_final.mol2 and decoys_final.mol2 were obtained from http://dude.docking.org/targets/cxcr4 [20]. Similar to CXCR4 (see Section 4.2.4), the whole crystal structure was employed in this study. The subsequent procedure followed the generic procedure (see Section 4.2.1). During the PLIF identification using PyPLIF HIPPOS, it was recognized that one active compound, CHEMBL163087, could not produce docking results. Apparently, AutoDock Vina did not proceed with the docking simulations for the Si-containing compound CHEMBL163087. The compound CHEMBL163087 was then annotated as a false negative in the subsequent procedure.

## 5. Conclusions

The combination of retrospective SBVS campaigns, PLIF-derived ensPLIF descriptors using PyPLIF HIPPOS, and RPART analyses provide a full in silico complementary method to SDM studies for the molecular determinants of the ligand binding to the corresponding GPCRs. Notably, the method shows better prediction quality indicators of the SBVS protocols compared to the original protocols. Moreover, for a particular receptor target, there are options to optimize the prediction quality, e.g., fine-tuning the configuration of the docking simulations or filtering poses prior to ensPLIF calculation based on the docking score.

## Figures and Tables

**Table 1:** The prediction quality of the retrospectively validated structure-based virtual screening (SBVS) protocols and the molecular determinants of the ligand binding to the studied receptors.

Receptor	SBVS Prediction Quality		Molecular Determinant		
	EF ^1^	F-Measure ^2^	Residue	Interaction Type ^3^	Retrospective Verification ^4^
AA2AR	272.286	0.184	Glu169	ionic (protein as anion)	verified
			Trp246	aromatic edge-to-face	verified
			Leu249	hydrophobic	verified
			His250	aromatic edge-to-face	verified
ADRB2	465.151	0.307	Trp109	aromatic edge-to-face	n.a. ^5^
			Asp113	ionic (protein as anion)	verified ^6^
			Asp192	hydrophobic	n.a. ^5^
			Phe193	aromatic face-to-face	n.a. ^5^
			Ser204	h-bond (protein as donor)	verified
			Trp286	hydrophobic	n.a. ^5^
			Phe289	aromatic edge-to-face	n.a. ^5^
			Phe290	aromatic edge-to-face	n.a. ^5^
			Asn312	h-bond (protein as donor)	verified
CXCR4	n.d. ^7^	0.333	Glu32	hydrophobic	verified
			Asp97	hydrophobic	verified
			Trp102	aromatic edge-to-face	n.a. ^5^
			Tyr255	aromatic edge-to-face	verified
DRD3	455.652	0.169	Phe106	hydrophobic	n.a. ^5^
			Val107	hydrophobic	n.a. ^5^
			Asp110	ionic (protein as anion)	verified ^6^
			Ile183	hydrophobic	verified
			Phe346	aromatic edge-to-face	n.a. ^5^
			His349	aromatic edge-to-face	verified

^1^ Enrichment Factor (EF) = true positive rate/false positive rate; ^2^ F-measure = (2 × recall × precision)/(recall + precision) [21]; ^3^ refers to [16,18]; ^4^ refers to GPCRdb [5,11]; ^5^ n.a. = not available; ^6^ a conserved residue in aminergic GPCRs; ^7^ n.d. = not determined since the false positive rate value was 0.

## Data Availability

The data presented in this study are available in this article as Supplementary Materials.

## Supplementary Materials

The following are available online, Table S1: Table-S1-GPCR-ensplif.zip.

## Sample Availability

Not available.
